# Imagine HEALTH: results from a randomized pilot lifestyle intervention for obese Latino adolescents using Interactive Guided Imagery^SM^

**DOI:** 10.1186/1472-6882-14-28

**Published:** 2014-01-17

**Authors:** Marc J Weigensberg, Christianne J Lane, Quintilia Ávila, Kati Konersman, Emily Ventura, Tanja Adam, Zohreh Shoar, Michael I Goran, Donna Spruijt-Metz

**Affiliations:** 1 Department of Pediatrics, University of Southern California Keck School of Medicine, 2250 Alcazar St, Ste 211, Los Angeles 90089, CA, USA; 2 Department of Preventive Medicine, University of Southern California Keck School of Medicine, Los Angeles, CA, USA

**Keywords:** Guided imagery, Obesity, Childhood, Latino, Adolescents, Lifestyle, Diabetes

## Abstract

**Background:**

There is an urgent need for innovative and developmentally appropriate lifestyle interventions to promote healthy lifestyle behaviors and to prevent the early onset of type 2 diabetes and cardiovascular disease risk in obese Latino adolescents. Guided imagery offers promise to reduce stress and promote lifestyle behavior change to reduce disease risk in obese adolescents. Our objectives were: 1) To pilot test a new 12-wk lifestyle intervention using a randomized trial design in obese Latino adolescents, in order to determine the effects of the mind-body modality of Interactive Guided Imagery^SM^ (IGI), over and above those of a didactic lifestyle education, on insulin resistance, eating and physical activity behaviors, stress and stress biomarkers; and 2) To explore the role of intervention-related changes in stress and stress biomarkers on changes in metabolic outcomes, particularly insulin resistance.

**Methods:**

Obese (BMI > 95^th^ percentile), Latino adolescents (n = 35, age 14-17) were randomized to receive either 12 weekly sessions of a lifestyle education plus guided imagery program (GI), or lifestyle education plus a digital storytelling computer program (DS). Between-group differences in behavioral, biological, and psychological outcomes were assessed using unpaired T-tests and ANCOVA in the 29 subjects who completed the intervention.

**Results:**

The GI group demonstrated significant reductions in leisure sedentary behavior (p < .05) and increases in moderate physical activity (p < .05) compared to DS group, and a trend toward reduced caloric intake in GI vs DS (p = .09). Salivary cortisol was acutely reduced by stress-reduction guided imagery (p < .01). There were no group differences in adiposity, insulin resistance, perceived stress, or stress biomarkers across the 12-week intervention, though decrease in serum cortisol over the course of the intervention was associated with improved insulin sensitivity (p = .03) independent of intervention group and other relevant co-variates.

**Conclusions:**

The improvements in physical activity and stress biomarkers following this pilot intervention support the role of guided imagery in promoting healthy lifestyle behavior change and reducing metabolic disease risk in obese Latino adolescent populations. Future investigations will be needed to determine the full effects of the Imagine HEALTH intervention on insulin resistance, stress, and stress biomarkers.

**Trial registration:**

Clinicaltrials.gov Registry #: NCT01895595

## Background

The prevalence of obesity and obesity-related disorders, such as type 2 diabetes (T2D) and the constellation of cardiovascular disease (CVD) risk factors known as the metabolic syndrome among adolescent Latinos has increased dramatically in recent years [1]. Latinos have a 50% lifetime risk of developing diabetes [2], raising the specter of significant early morbidity in mid-adulthood, as well as the potential for enormous increases in societal health care expenditures [3]. Results from our prior studies demonstrate that there is a high prevalence of pre-diabetes (~32%) [4-6], and metabolic syndrome (~30%) [7] in overweight/obese Latino youth, and that Latino youth with either persistent pre-diabetes or persistent metabolic syndrome show deteriorating pancreatic islet beta-cell function over time, suggesting progression toward development of T2D [8,9]. Finally, Latino youth with persistent metabolic syndrome have higher carotid artery intima media thickness, a strong marker for future atherosclerotic disease [10,11]. We have concluded from these studies that insulin resistance, rather than body fat *per se*, is the primary patho-physiological factor leading to metabolic disease risk in youth as it is in adults [3,12].

Reducing insulin resistance through lifestyle intervention therefore holds promise to prevent the early onset of T2D and CVD in obese adolescents. However, past obesity intervention strategies in adolescents have had a number of limitations including limited studies of minority, economically disadvantaged populations; emphasis on adiposity outcomes that make it difficult to assess the broader physiological impacts that interventions can claim (i.e. effects on insulin resistance), reliance on cognitive models of human thinking and behavior, which may be developmentally suboptimal for adolescents [13,14]; and lack of attention to factors beyond eating and physical activity that may be promoting obesity-related disease, such as stress.

There is a considerable literature implicating stress as a factor in obesity-related disease risk. Stress promotes obesity through its effects on eating behaviors, resulting in a preference for the ingestion of higher calories and more calorically dense snack foods [15-18]. Studies in primates [19] and human adults[20-23] also suggest that chronic stress, via neuroendocrine mechanisms producing hypothalamic-pituitary-adrenal (HPA) axis activation and subtle hypercortisolism, can result in a “pseudo-Cushingoid” obesity phenotype characterized by visceral adiposity, insulin resistance, and metabolic syndrome [22,24-27]. We have shown support for this concept in overweight Latino adolescents, whereby higher morning serum cortisol levels are associated with metabolic syndrome [28], and predict future deterioration of insulin sensitivity [29].

The role of stress in obesity-related complications is made particularly relevant since modern day adolescents suffer from increased anxiety relative to those of the past [30], suggesting that today’s children may be exposed to increased numbers of stressors than in the past. Minorities also experience higher levels of stress [31], while socioeconomic and immigration-related factors may constitute additional stressors in Latinos [32]. In addition, the high school years are a particularly vulnerable time [33], and 25.3% of high school students in Los Angeles report being “very stressed” on a daily basis [34]. Thus, inner city Latino teens are a population for whom stress influences on obesity-related complications may be particularly relevant.

Stress-reduction using mind-body modalities may therefore represent a promising approach for use in adolescent obesity interventions. This is supported by findings that mind body therapies such as hypnotherapy and mindfulness approaches may be useful in obesity treatment in adults [35-37]. Guided imagery is a mind-body healing modality which involves the generation of mental images for the purpose of achieving specific health outcomes, such as evoking a state of relaxation or mobilizing immune system response [38]. It is also a powerful stress management technique, similar to related mind-body therapies that have been shown to reduce stress, stress-related symptoms [39], and acutely reduce salivary cortisol levels [40,41]. In addition to stress reduction, guided imagery and other related mind-body interventions have also been used to improve health behaviors, specifically including eating behaviors in eating disorders [42,43]. Guided imagery therefore has the potential to both reduce stress and improve lifestyle behaviors, making it a promising approach to adolescent obesity interventions. To date however, there are no reports on the effects of guided imagery or other mind-body interventions on insulin resistance, stress biomarkers, or lifestyle behaviors in obese adolescents.

Given the above considerations, the objectives of this study were:

1. To pilot test a new 12-wk lifestyle intervention using a randomized controlled design in obese Latino adolescents, in order to determine the effects of the mind-body modality of Interactive Guided Imagery^SM^, over and above those of a didactic lifestyle education, on insulin resistance, eating and physical activity behaviors, stress, and stress biomarkers (serum, urinary, and salivary cortisol).

2. To explore the role of intervention-related changes in stress and stress biomarkers on changes in metabolic outcomes, particularly insulin resistance.

We hypothesized that participants who received the didactic lifestyle curriculum plus the guided imagery program would show greater improvements in insulin resistance, physical activity, dietary intake, and stress, as compared to those receiving the didactic lifestyle education as the only intervention. We further hypothesized that intervention-related reductions in stress biomarker exposure would predict improvements in insulin sensitivity, independent of adiposity.

## Methods

### Intervention program

We utilized an iterative process involving focus groups, preliminary program testing, and idea-building groups to develop the 12-week Imagine H.E.A.L.T.H. (**H**ealthy **E**ating **A**ctive **L**iving **T**otal **H**ealth) lifestyle intervention, which contained 2 main components: didactic lifestyle education and guided imagery. All lifestyle lesson plans and guided imagery scripts developed during this phase of the project were contained within lesson binders for use by the health educators and guided imagery facilitators in conducting the subsequent RCT.

### Lifestyle education component

The didactic lifestyle education curriculum was based on 2 major components: 1) intuitive eating; and 2) modification of dietary carbohydrate intake. Intuitive eating is a non-dieting philosophy which emphasizes five major principles: 1) unconditional permission to eat when hungry, and to eat whatever food is desired; 2) eating for physical rather than emotional reasons; 3) reliance on internal hunger and satiety cues to determine when and how much to eat; 4) seeking satisfaction in eating; and 5) incorporating movement/physical activity to promote physical well-being [44-46]. Intuitive eating supports autonomy in eating and physical activity decision-making, and is therefore a developmentally ideal paradigm for adolescents, for whom the pursuit of autonomy is a critical developmental milestone [47]. The rationale for education about modifying the quality of carbohydrate intake, i.e. increasing whole grain and fiber while reducing refined and added sugar intake, relates to the consistent link between increased sugar-sweetened beverage consumption and poor metabolic health [48]. Additionally, intervention studies in obese adolescents by our group and others have shown improvement in body fat and/or insulin resistance resulting from such dietary carbohydrate moderation [49-51]. The 12-week lifestyle lesson plan (Table 1), contained 3 lessons covering the principles of intuitive eating, and 3 lessons covering general healthy nutritional information, including modification of carbohydrate intake [52]. A personal trainer led 2 lessons covering “Active Living”, movement and physical activities or exercises which participants could easily learn and do in their own homes using inexpensive, generally available materials such as water-filled plastic containers. Subsequent lessons addressed environmental and internal obstacles to behavior change, the effect of emotional eating on overall eating habits, and the role that self esteem and body image play in impacting health choices. The final session integrated all prior lessons.

**Table 1:** Imagine HEALTH 12-week curriculum

**Session**	**Lifestyle curriculum**	**Guided imagery curriculum**
1	Introduction to HEALTH	Stress-reduction 1: Focused breathing and muscle relaxation
2	Diets don’t work	Stress-reduction 2: Relaxed place image
3	Honor your hunger, Respect your fullness	Stress-reduction 3: Conditioned relaxation
4	Active Living 1: Benefits of physical activity	Healthy Eating 1: Fullness symbol image
5	Active Living 2: Your personal balance	Active Living 1: Physical activity image
6	Healthy Eating 1: Nutrition basics	Active Living 2: Personal meanings of physical activity
7	Healthy Eating 2: Fiber and healthy grains	Healthy Eating 2: Healthy Eating image
8	Healthy Eating 3: Added sugars	Healthy Eating 3: Personal meanings of healthy eating
9	Healthy habits in a toxic environment	Resistance 1: Working with resistance to healthy eating
10	Emotional and distracted eating	Resistance 2: Transforming unhealthy habits
11	Respect your body	Looking Ahead 1: Ideal model image
12	Integration: Total HEALTH	Looking Ahead 2: The next step

### Interactive Guided Imagery^SM^ component

Scripts for twelve 45-minute guided imagery sessions were modified by the lead author from those provided by our expert consultant. Using established methods of Interactive Guided Imagery^SM^ (IGI) [53], each session consisted of 10-15 minutes of “foresight” (discussion of the upcoming imagery exercise), 20 minutes of “insight” (the actual imagery exercise), and 10-15 minutes of “hindsight” (discussion and debriefing of the imagery experience). As opposed to other forms of guided imagery, IGI does not utilize rote scripts to generate the same image in all subjects. Instead, it uses standardized, yet adaptable, techniques designed to enable the subject to engage his/her own personal, subjective images relating to a given topic in order to develop health-directed insights, health promoting behavior changes, or produce direct physiological changes. For example, during stress-reduction IGI, rather than suggesting a presumably relaxing standard image such as a calm beach at sunset, the imagery facilitator would suggest that the subject invite an image of a place that represents just comfort or relaxation. The facilitator then guides the subject to deeply engage that image using sensory recruitment, dialogue with the image, or kinesthetic amplification, all the while maintaining continuous dialogue and communication with the subject in order to maximize the affective experience.

Specific imagery content of the first 3 IGI sessions (Table 1) consisted of stress reduction imagery: Session 1) slow, focused breathing combined with progressive muscle relaxation; Session 2) “relaxed place” imagery - an imaginal exploration of a place that represented only comfort, safety, and relaxation; Session 3) “conditioned relaxation” [54] following another “relaxed place” imagery experience, a “signal breath” was used to rapidly induce a conditioned relaxation response. Session 4 explored personal images and sensations related to fullness and hunger. Sessions 5 and 7 allowed participants to explore images of themselves actually being physically active and eating in a healthy way. Sessions 6 and 8 utilized the standard IGI technique of “Inner Advisor” imagery, in which participants dialogued with a beneficent inner figure that offered guidance to help them understand the personal meanings that eating healthily and being physically active hold for them, and provide reasons to improve lifestyle habits. For Sessions 9 and 10, participants dialogued with an image of their “Inner Warrior”, an adapted form of the Inner Advisor who had the additional qualities of strength, discipline, courage, and self-confidence, in order to work through resistance and obstacles (both internal and external) to healthy eating and physical activity behaviors. Finally, Sessions 11 and 12 explored images of what future life might be like if participants were to consistently follow healthy eating and active living practices, and what specific next steps they could take to move toward that outcome.

To control for the potential confounder of staff contact time, participants not randomized to IGI received an alternative digital-storytelling program (DS) that was adapted from the MOSAIC after school program in East Oakland, CA (http://mosaicguide.blogspot.com/). This consisted of 12 weekly, individualized, 45-minute sessions which taught computer and digital video/photography techniques with which participants created and edited a short digital film or story. Participants chose their own topics, unrelated to obesity or health issues. A non-treatment control arm was not included due to budgetary constraints of this small pilot study, and because our main aim was to isolate the effects of IGI over and above the didactic lifestyle education.

### Randomized controlled pilot intervention (RCT)

The intervention was piloted in obese (BMI greater than 95^th^ percentile for age and sex), Latino (by parental self-report) adolescents in Los Angeles, CA, aged 14-17 years, in 9th grade or higher, recruited from pediatric clinics, health fairs, or word of mouth. Prospective participants were screened by trained research assistants using a checklist to determine the presence of any exclusion criteria, including participation in any weight loss program within the previous 6 months, serious chronic illness or any medical condition (e.g. diabetes, hypothyroidism) or medication (e.g. prednisone) that would affect body composition or insulin sensitivity, undiagnosed diabetes (ruled out using a standard outpatient oral glucose tolerance test ~2 weeks before onset of intervention), regular participation in any mind-body stress reduction or related practices in the past, clinically diagnosed psychiatric or eating disorder, or impairment in cognition, language, or hearing. This study was approved by the Institutional Review Board of the University of Southern California (USC). Written parental consent and youth assent were obtained prior to initiation of any study procedures.

**Figure 1:**
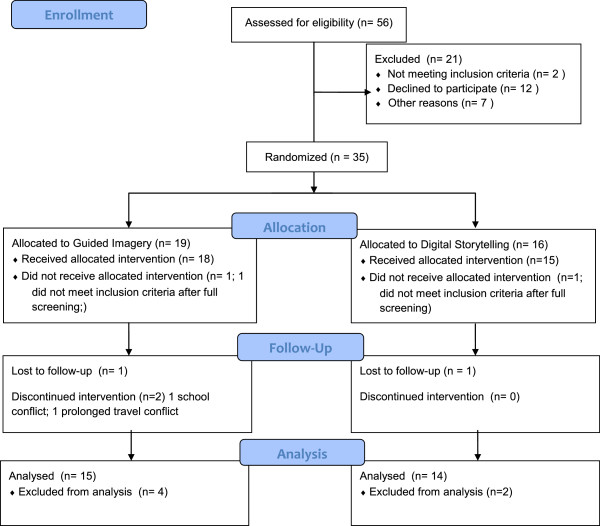
Consort flow diagram.

A stratified, block randomization schema generated by the study statistician (co-author CJL) was used to ensure gender balance in the 2 study groups: Lifestyle education + Guided imagery (GI) versus Lifestyle Education + Digital Storytelling (DS). Intended recruitment for this pilot trial was set at 40 (20 per group), based on logistical and funding limits, along with a power analysis based on the large change in insulin sensitivity seen in our resistance training (i.e. weight-lifting) pilot intervention [55]. The recruitment goal was not fully reached due to the need to stop recruitment and begin the intervention in time to allow completion of the intervention before interruption by school vacations. Of the 35 participants randomized (Figure 1), 2 were lost to follow-up before starting the intervention, 3 in GI and 1 in DS did not complete the intervention and did not obtain final outcome measures, and 29 completed the entire intervention including final outcome measures. Both groups received the same Imagine HEALTH didactic lifestyle education curriculum in separate classes for each intervention group, taught weekly by trained health educator staff for 45 minutes between 5-7 PM in a conference room setting, with 4-6 participants per class. Classes were either preceded or followed immediately by either 45-minute IGI sessions (GI Group), or by 45-minute digital storytelling sessions (DS Group). Guided imagery was delivered one-on-one to participants by certified IGI practitioners (co-authors MW, DSM, or staff), in a quiet counseling room setting, while DS sessions were delivered one-on-one by trained project staff in a classroom or office setting. Transportation was provided to all participants as needed (reimbursement for public transportation, taxis). Make-up sessions were offered if either lifestyle class or guided imagery/digital storytelling sessions were missed. Participants in the GI group were encouraged to practice the imagery at home for 5-10 minutes twice a day. Participants were asked at the beginning of each weekly session whether or not they were practicing their imagery exercises at home, but practice was not formally assessed.

### Pre- and post-intervention assessments

Participants were admitted to the USC General Clinical Research Center (GCRC) in the late afternoon. Vital signs and anthropometric measures were taken, followed by a physical exam by a licensed pediatric care provider. Perceived stress survey was administered, followed by an overnight fast, with only water permitted after 8 PM. Urine was collected from 10 PM to 6 AM to determine urinary free cortisol. Saliva was collected at 9:30 PM, at 5:30 AM the following morning when awakened by nursing staff, and at 6 AM, using the Salivette system (Sarstedt, Newton, NC). Participants placed a cotton swab in the mouth for 2 minutes without chewing to passively absorb saliva. Saliva samples were processed immediately by centrifuging at 2500 rpm for 10 minutes and frozen at -70°C until assayed. Fasting blood was drawn to assess morning serum cortisol, followed by a 3-hour insulin-modified, frequently-sampled intravenous glucose tolerance test (FSIVGTT), as previously described [5].

### Outcome measures

#### Program acceptability outcomes

Acceptability of the intervention was assessed by qualitative analysis of post-intervention focus group responses, from the frequency of class attendance, and from subjective surveys during the RCT. For the latter, upon completion of each lifestyle class and each guided imagery session, participants responded to the prompt, “Overall how much did you like this session?” on a Likert scale ranging from 1 (“Did not like at all”) to 10 (“Very much liked”).

#### Behavioral outcomes

Physical activity was assessed using the 3-Day Physical Activity Recall (3-DPAR), a self-report measure which provides reports of physical activity over three consecutive days and has been validated against accelerometry in youth [56]. Participants identified activities from a 71-item list for each half hour for two weekdays and one weekend. Activity types were converted into light, moderate, or vigorous physical activity using the compendium of physical activities [57] and Freedson’s cutpoints for youth [58]. Half-hour blocks spent watching television/movies, playing video games/surfing the internet, and talking on phone were coded separately as leisure sedentary behaviors. Mean minutes spent at each activity level were obtained by averaging total minutes across 3 days. Records were adequately completed in 24 participants.

Dietary intake was assessed using 3-day diet records and analyzed as previously described [59]. Briefly, trained staff instructed participants to accurately record their dietary intake for 2 weekdays and 1 weekend day and clarified the diet records in detail with participants upon completion. Data were entered into the NDS-R software program (version 5.0_35) for nutrient analysis and checked for accuracy. Data from the 3 days were averaged to calculate daily intake of energy, macronutrients, total and added sugars, and fiber. Records were adequately completed in 23 participants.

Intuitive Eating was assessed using Hawks’ Intuitive Eating Scale, a 27-item, 4-point Likert scale survey [45] that consists of 4 subscales: 1) Intrinsic eating (4 items), relating to reliance on physical cues to start and stop eating; 2) Extrinsic eating (6 items), relating to avoidance of emotional or external prompts for eating decisions; 3) Anti-dieting (13 items) measuring disagreement with dieting behaviors; and, 4) Self-care (4 items), assessing an orientation that favors health & fitness over fashion or beauty. Higher scores for all subscales and total score indicate more intuitive eating.

Stress was assessed using the Perceived Stress Scale (PSS) [60], a 14-item survey that inquires about perception of stressful experiences in the preceding month. As previously reported, this measure was revised for comprehension and three items were added based on interviews with urban Latino youth [61].

#### Biological outcomes

Weight and height were measured in triplicate using a beam medical scale and wall-mounted stadiometer, to the nearest 0.1 kg and 0.1 cm, respectively. BMI percentile and Z-score were calculated using EpiInfo, Version 3.3.2 software. Tanner pubertal stage was assigned based on breast stage for girls and pubic hair stage for boys. Percent body fat was determined after at least a 3-hour fast by air displacement plethysmography (Bod Pod® Life Measurement Instruments, Concord, CA). FSIVGTT values for plasma glucose and insulin were entered into the MINMOD MILLENIUM 2003 computer program (Version 5.16, Richard N. Bergman, USC) [62] to determine insulin sensitivity. HOMA-insulin resistance index was calculated [63]: HOMA-IRI = Fasting insulin (μU/ml) × Fasting glucose (mmol/L)/22.5. Diurnal HPA activity was assessed by measurement of overnight urinary free cortisol, fasting morning serum cortisol, the nocturnal cortisol rise (NCR), (increase in salivary cortisol from bedtime to awakening), and salivary cortisol awakening response (CAR), (increase in salivary cortisol between 5:30 AM and 6 AM) [64]. In addition, the acute salivary cortisol response to stress-reduction guided imagery was determined as the difference from the beginning to the end of each of the first 3 weekly 45-minute guided imagery or digital storytelling sessions.

### Assays

Plasma insulin, salivary cortisol, and serum cortisol were assayed using an automated enzyme immunoassay (Tosoh AIA 600 II analyzer, Tosoh Bioscience, Inc, South San Francisco, CA). Insulin assay sensitivity was 0.3 μU/ml, inter-assay CV 5.8% and intra-assay CV 2.9%. Serum and salivary cortisol assay sensitivity was 0.02 μg/dl, inter-assay CV 11.2% and intra-assay CV 8.2%, as previously reported [65]. Urinary free cortisol was assayed by Coat-A-Count RIA (Diagnostic Products, Los Angeles, CA), with correction of the total overnight urinary free cortisol for body surface area (μg/total volume/m^2^). Glucose was assayed by Yellow Springs Instrument 2700 Analyzer (YSI Inc, Yellow Springs, OH, USA).

### Statistical analyses

Data were analyzed for the 29 participants who completed the full intervention, 14 in DS group and 15 in GI group. Descriptive statistics were performed for all baseline variables. Normality was assessed on continuous variables, and log transformation performed *a priori* on all non-normally distributed variables (insulin sensitivity, HOMA, waist circumference). Two individual outliers > 3 SD from the group means were excluded from analyses (these included baseline values in one subject each for insulin sensitivity in GI group, and for urinary free cortisol in DS group). Missing data included baseline waist circumference not obtained in one subject in each group, and several salivary cortisol samples in each group with quantity insufficient for analysis. Intervention group differences in unadjusted baseline variables were assessed using t-tests (continuous variables) or chi square tests (categorical variables). Unadjusted change scores in variables of interest were calculated and between-group differences assessed by unpaired t-tests. In addition, intervention effects on changes in physical activity and dietary intake were analyzed by ANCOVA, with intervention group as main factor, adjusting *a priori* for baseline value. Because this was a pilot study, and the statistics were anticipated to be underpowered, effect sizes (Cohen’s ∆) were computed to indicate degree of clinical relevance to the results. Linear regression models were used to explore the degree to which changes in the dependent variable insulin sensitivity could be attributed to changes in the individual measures of HPA activity or perceived stress, adjusting *a priori* for age, sex, percent body fat, intervention group, baseline insulin sensitivity, and HPA measure at baseline. All analyses were carried out using SPSS, version 16.0, with significance set at α = .05.

## Results

### Baseline group comparisons

Baseline characteristics of the 6 participants who did not complete the intervention were not statistically different from those who did complete the intervention in terms of age, sex, pubertal status, adiposity, or insulin resistance, indicating that the evaluable sample was representative of the whole. There were no differences at baseline between DS vs GI group in age (16.1 ± 0.95 vs 15.5 ± 1.0 years), sex (7 M/7 F vs 7 M/8 F), or pubertal stage (14 in each group ≥ Tanner stage 4, 1 boy Tanner stage 2 in GI group). There were also no group differences in baseline adiposity, physical activity, dietary intake, perceived stress, HPA/stress biomarkers, or metabolic measures (left-hand columns of Tables 2 and 3), which demonstrated an obese and insulin resistant study population.

**Table 2:** Lifestyle behaviors: baseline values and change with intervention

**Outcome variable**	**Lifestyle + DS**		**Lifestyle + GI**	
	**Baseline**	**Change**	**Baseline**	**Change**
Physical activity		(n = 12)		(n = 12)
Sedentary-leisure (min/d)	132.1 ± 59.3	72.9 ± 145.8	173.8 ± 122.6	-65.4 ± 124.0*
Moderate (min/d)	115.8 ± 101.5	-60.8 ± 122.6	103.8 ± 73.5	30.4 ± 102.0^†^
Moderate-vigorous (min/d)	171.7 ± 111.0	-48.3 ± 155.4	149.2 ± 106.5	15.0 ± 131.7
Vigorous (min/d)	55.8 ± 46.0	12.5 ± 72.0	44.2 ± 62.6	-14.2 ± 72.3
Dietary intake		(n = 10)		(n = 13)
Energy (kcals/d)	1715 ± 555	142.6 ± 623.9	1892 ± 584	-277.3 ± 512.4^†^
Carbohydrates (% kcals)	51.4 ± 9.2	.77 ± 11.0	49.5 ± 10.0	-1.44 ± 8.0
Fat (% kcals)	31.9 ± 8.7	.85 ± 9.7	33.0 ± 8.5	.83 ± 7.0
Protein (% kcals)	16.7 ± 4.2	-1.60 ± 5.71	17.5 ± 3.8	.52 ± 5.09
Total sugars (% kcals)	23.1 ± 6.1	-1.34 ± 9.51	22.9 ± 9.4	-2.46 ± 8.02
Added sugars (% kcals)	18.0 ± 6.8	-1.92 ± 10.6	13.5 ± 7.8	-.70 ± 7.99
Total fiber (g/1000 kcal)	8.27 ± 3.65	-.08 ± 5.45	7.89 ± 4.37	.64 ± 4.63
Soluble fiber (g/1000 kcal)	2.52 ± 1.21	.17 ± 2.26	2.60 ± 1.32	-.02 ± 1.40
Insoluble fiber (g/1000 kcal)	5.65 ± 2.65	-.43 ± 3.65	5.12 ± 3.25	.75 ± 3.70
Intuitive eating				
Total score	2.72 ± 0.50	0.15 ± 0.29	2.85 ± 0.41	0.32 ± 0.36*
Intrinsic eating	2.20 ± 0.32	0.13 ± 0.50	2.25 ± 0.70	0.21 ± 0.98
Extrinsic eating	3.05 ± 0.82	-0.22 ± 0.41	3.13 ± 0.66	0.23 ± 0.65*
Anti-dieting	2.81 ± 0.63	0.28 ± 0.41	3.07 ± 0.47	0.34 ± 0.40
Self-care	2.45 ± 0.53	0.33 ± 0.62	2.30 ± 0.56	0.53 ± 0.97*

Data show Mean ± SD for baseline and unadjusted change from pre- to post-intervention. Positive direction to change indicates more physical activity, more sedentary behavior, more dietary intake, and more intuitive eating.

(n) indicates number of comparisons made when complete group data (n = 14 for DS group and n = 15 for GI group) not available.

Between group comparisons by unpaired T-tests: *p < .05 ^†^p < 0.1.

**Table 3:** Adiposity, insulin resistance, and stress outcomes: baseline values and change with intervention

**Outcome Variable**	**Lifestyle + DS**		**Lifestyle + GI**	
	**Baseline**	**Change**	**Baseline**	**Change**
Adiposity				
BMI (kg/m^2^)	35.1 ± 4.6	0.16 ± 1.37	36.2 ± 5.9	0.77 ± 1.18
BMI-Z score	2.23 ± 0.22	-0.01 ± 0.13	2.30 ± 0.4	0.04 ± 0.09
% Body fat	37.8 ± 8.4	0.39 ± 5.3	41.5 ± 7.4	0.78 ± 4.0
Waist circumference (cm)	111.6 ± 10.3 (13)	-0.77 ± 3.2 (13)	118.1 ± 13.6 (14)	0.64 ± 1.6 (14)
Insulin sensitivity/Resistance				
Insulin sensitivity (×10^-4^ /(μU/ml)/min)	1.74 ± 0.93	0.43 ± 0.69	1.28 ± 0.65 (14)	-0.05 ± 0.65^†^ (14)
HOMA-IRI	4.02 ± 3.36	-0.86 ± 1.7	5.20 ± 2.30	-0.78 ± 2.1
Stress/HPA axis				
Perceived stress	29.6 ± 8.9	0.07 ± 7.68	29.8 ± 6.6	-2.00 ± 7.13
Serum cortisol (μg/dl)	13.3 ± 4.5	-0.89 ± 5.84	11.0 ± 4.2	0.64 ± 3.99
Urine free cortisol (μg/m^2^/8 hrs)	3.20 ± 1.44 (13)	1.71 ± 3.15 (13)	3.98 ± 2.05	-0.57 ± 3.70^†^
Nocturnal cortisol rise (μg/dl)	0.66 ± 0.56 (12)	0.67 ± 1.26 (9)	0.88 ± 0.42 (9)	-0.24 ± 0.42^†^ (9)
Cortisol awakening response (μg/dl)	1.03 ± 0.47 (11)	-0.68 ± 1.17 (9)	0.69 ± 0.56 (13)	0.03 ± 0.70^†^ (13)

Data show Mean ± SD for baseline and unadjusted change from pre- to post-intervention.

Between group comparisons by unpaired T-tests: *p < .05 ^†^p < 0.1.

(n) indicates number of comparisons made when complete group data (n = 14 for DS group and n = 15 for GI group) not available.

### Program acceptability outcomes

Attendance rates were high in both groups (90-100% of all education, IGI, and DS sessions), and there was a high level of acceptability by the participants to all components of the intervention as determined by post-intervention focus groups and self-report evaluations. Example comments from focus group participants demonstrated that: 1) though the concept of GI was somewhat perplexing at the start, it was ultimately accepted: “*Yeah*, *I got into it*, *you know* . . . *but in the beginning it was kind of weird* . . . “; 2) stress reduction imagery was very useful, even beyond the bounds of the study: “*It helped me when I was stressed out in school. I just started doing it and I would feel better*.”; 3) using imagery of a “fullness symbol” to recognize satiety was particularly useful: “*It has been helping me. Now I actually stop* (*eating*) *when I*’*m full. Before it would be like*, *oh*, *blah blah blah*, *so I continued to eat. Now I just stop*.”; 4) active living imagery (i.e. image of oneself actually being physically active) led to insights that could potentially motivate behavior change: “*I liked it. My physical activity was surfing. I*’*m going to tell my brother to teach me*.” Despite the apparent enjoyment of the imagery process, there was almost universal non-adherence with the recommendation to practice imagery exercises at home between imagery sessions, with reported home practice erratic at best. As determined by self-report questionnaires, there was a high level of acceptability by the participants to all components of the intervention, with acceptability scores consistently between 9-10 out of 10 for all 12 lifestyle classes, as well as for all 12 guided imagery sessions.

### Intervention outcomes

**Figure 2:**
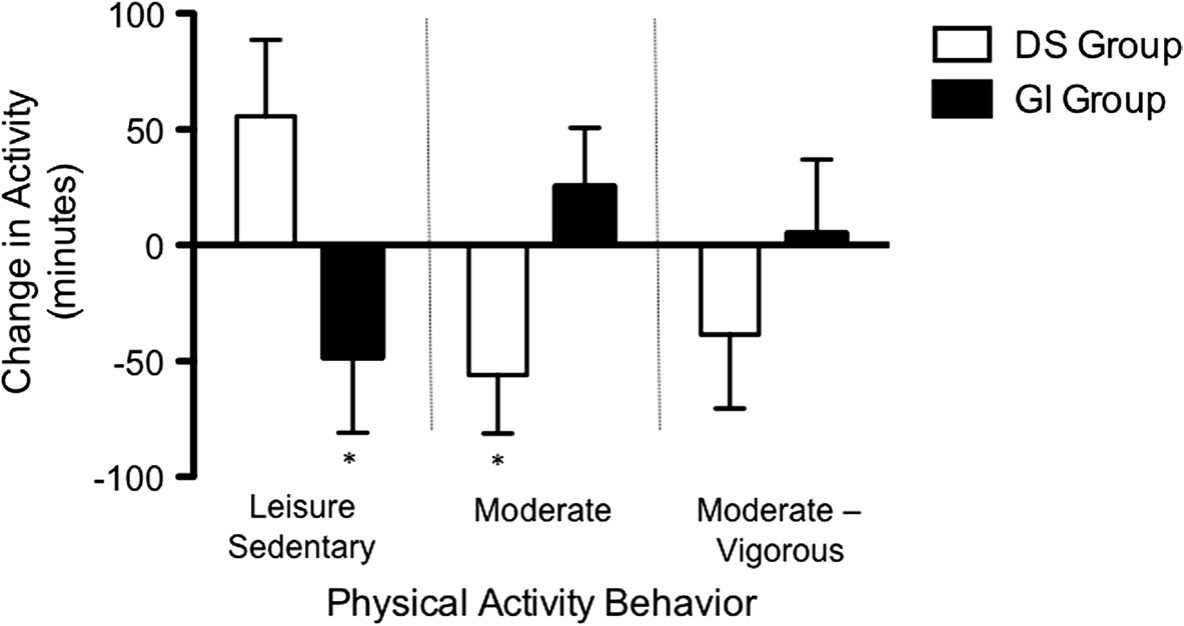
**Changes in sedentary behavior and physical activity.** Change from pre- to post-intervention sedentary and physical activity, adjusted for baseline values. *p < .05.

Baseline and post-intervention changes in sedentary and physical activities are shown in Table 2 (unadjusted), showing no differences in activity levels at baseline and a decrease in sedentary activity after intervention in the GI group (p < .05). After adjusting for baseline activity, sedentary behavior was significantly decreased by ~38% (p < .05, Δ = 1.0), and moderate physical activity was significantly increased by ~29% (p<.05; Δ = 0.8) in the GI group compared to the DS group (Figure 2). Changes between the 2 groups in vigorous or moderate-vigorous activity were not significant. There were no significant between-group differences in dietary total sugar or added sugar intake, either at baseline or post-intervention (Table 2). The non-significant decrease in total daily energy intake in the GI group compared to DS was of moderate effect size (Δ = 0.7; p = .09). The GI group showed significant increases in intuitive eating Total Score, as well as Extrinsic Eating and Self-care subscale scores.

**Figure 3:**
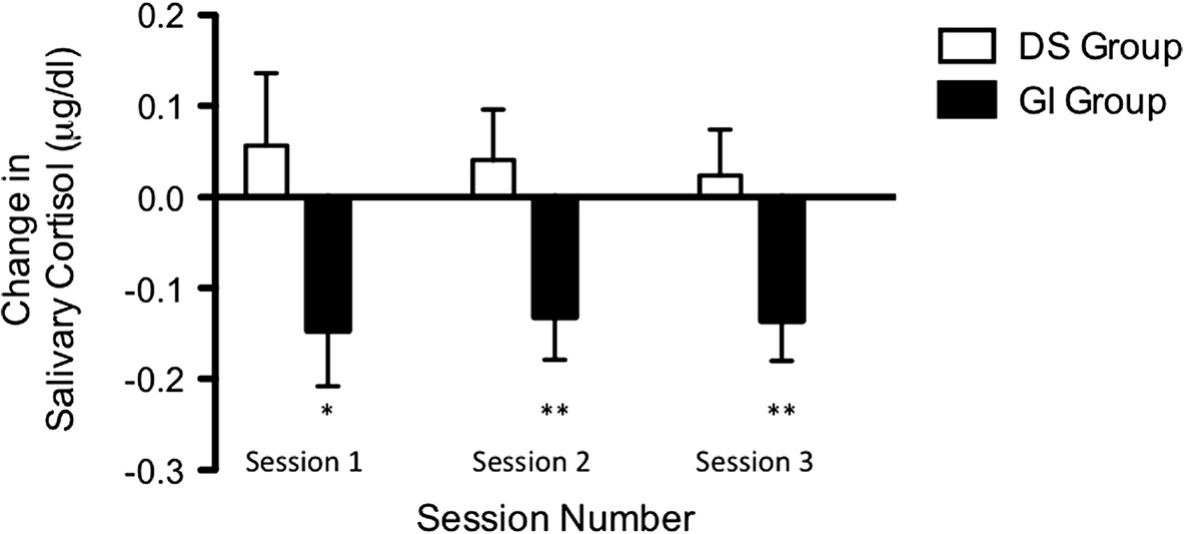
**Acute effects of stress-reduction Interactive Guided Imagery**^**SM **^**on salivary cortisol.** Change from pre- to post-session salivary cortisol, adjusted for pre-session values. *p = .05 **p < .05.

**Figure 4:**
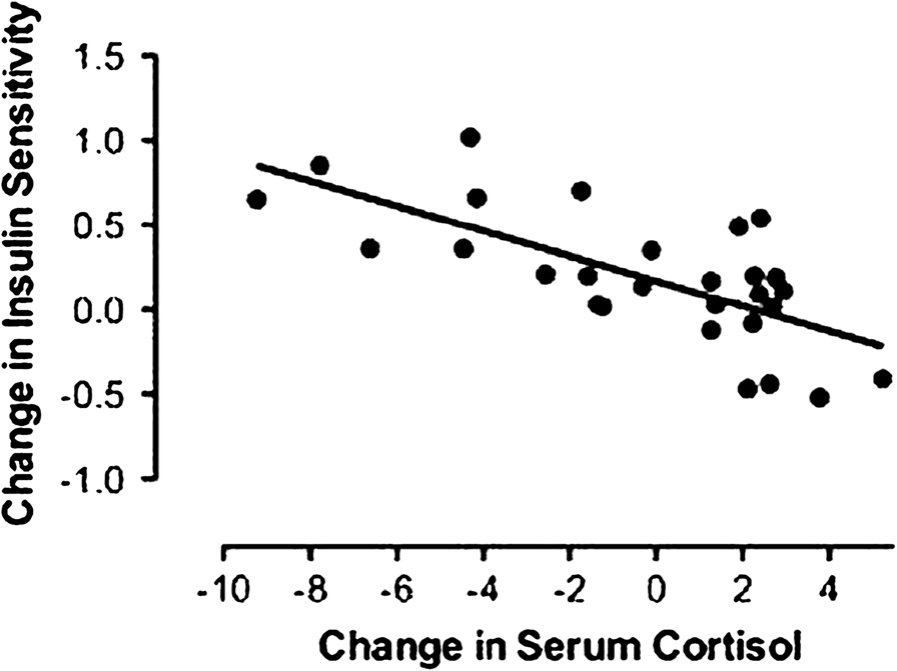
**Change in insulin sensitivity versus change in serum cortisol across the 12-week intervention.** Dependent variable: Change in insulin sensitivity. Independent variable: Change in serum cortisol. Covariates: Age, sex, intervention group, baseline percent body fat, change in percent body fat, baseline insulin sensitivity, baseline serum cortisol. Β_standardized_ = -0.60; R^2^ = .23; p = .03.

There were no significant between-group differences in changes in insulin sensitivity/resistance, adiposity, perceived stress, or any diurnal HPA measures across the 12-week intervention (Table 3). However, salivary cortisol was significantly reduced acutely across each of the three 45-minute guided imagery sessions that were specifically geared to stress-reduction (Figure 3). For these 3 stress-reduction sessions, guided imagery reduced salivary cortisol by an average of ~38%, compared to an ~8% increase in the DS group (p < .05, Δ = 3.0). Independent of intervention group, a decrease in serum cortisol over the course of the intervention was associated with an increase in insulin sensitivity (Figure 4; β_standardized_ = -.60, R^2^ = .23, p = .03) after controlling for age, sex, percent body fat, and baseline levels of dependent and independent variables. There were no other significant associations between changes in any of the other HPA or perceived stress measures and changes in insulin sensitivity across the intervention (data not shown).

## Discussion

In response to the pilot lifestyle intervention, our primary finding in this report was a reduction in leisure sedentary behavior and increase in moderate physical activity in the GI group as compared to the DS group. In addition, stress-reduction IGI produced large acute reductions in salivary cortisol across 45-minute sessions. There were no group differences in insulin resistance or adiposity measures seen as a result of the intervention. In addition, the Imagine HEALTH intervention was highly acceptable to this very at-risk obese Latino adolescent population, representing a critical first step in providing a new approach to obesity interventions in these youth. In particular, it was important to learn that IGI was accepted without any major difficulties in this population who were very naïve to mind-body complementary/alternative medicine modalities.

The large reduction in sedentary behavior and increase in moderate physical activity (effect sizes 0.8-1.0) in the IGI group represent substantial behavioral improvements, suggesting that the “Active Living” component of the IGI program successfully motivated behavior change. We speculate that having one’s own non-verbal, non-linear experience of physical activity through imagery, both mentally and kinesthetically, decreased resistance and increased acceptance to the idea of being physically active, ultimately leading to behavioral change. As a non-cognitive, affect-based process, guided imagery might be more developmentally appropriate and effective in motivating behavior change in adolescents than didactic, cognitive approaches, since adolescents’ thought processes may not always be logical, rational, or linear [13]. Thus the symbolic, affective content of images of seeing themselves being active, or receiving advice from the “Inner Advisor” and “Inner Warrior” figures on how to move past resistance to physical activity, may have led to internally motivated improvements in physical activity. While these results are encouraging, further study will be needed to fully elucidate the process by which IGI leads to increasing activity.

We found minimal intervention effect on the targeted sugar/fiber dietary intake behaviors. In contrast, there was evidence of an increase in the targeted intuitive eating behaviors in response to IGI, specifically a decreased reliance on external rules to guide eating (Extrinsic Eating) and an increase in orientation to health and fitness over appearance (Self-care). In addition, there was a trend of an overall reduction in caloric intake through the use of IGI. This is particularly notable, since the intuitive eating lifestyle intervention explicitly does not recommend caloric reduction in its promotion of healthy eating behaviors, but rather emphasizes healthy nutrition and physical activity principles with a non-diet (indeed anti-dieting) philosophy, seeking to restore healthy relationships to food and eating [44]. Consistent with adult findings showing cross-sectional relationships between intuitive eating practices and health markers [66,67], our findings suggest that intuitive eating can be safely used in obese adolescents without fear of sustained consumption of unhealthy foods. In other words, no sustained increases in calories, sugars or fats were seen despite the curriculum suggesting all foods are allowable, dieting is counterproductive, and portion size should be addressed using awareness of internal satiety and hunger signals rather than external rules.

The Imagine HEALTH stress-reduction component is thoroughly grounded in the literature implicating stress as a factor in obesity-related disease risk [21,24,26]. Stress-reduction IGI led to clear, acute reductions in salivary cortisol, which replicates our earlier findings in a smaller study group [68] and is comparable to reductions seen in other studies using similar mind-body modalities for stress-reduction [41], placing IGI in the company of those mind-body modalities that have a proven role in reducing stress and/or stress biomarkers, including in urban youth [69]. It would be interesting to conduct direct comparisons of the effectiveness of guided imagery to other stress-reduction mind-body modalities, such as mindfulness-based stress reduction [70], yoga [71], or hypnotherapy [35], which have been previously reported to have success in obesity interventions. Future work could also include prospective determination of subject hypnotizability [72] to determine the role this might play as a covariate in determining individual responses to guided imagery or other mind-body interventions. Beyond the acute lowering of cortisol with stress-reduction imagery, the lack of longer-term changes in stress or HPA markers across the full intervention may relate to the general noncompliance with home practice of stress-reduction imagery that participants reported. This may be reflective of the hectic home environments that many of our urban youth participants reported, including lower SES and multiple siblings and family members in one small household, with little time, space, and opportunity to practice the recommended imagery exercises. Alternatively, it is possible that our chosen outcome measures of perceived stress and HPA activity markers may not be subject to change even with consistent practice. In addition to limiting the effects on HPA activity, the lack of consistent home practice of the prescribed guided imagery may have limited its effectiveness in promoting the desired lifestyle behavior changes. Future studies with attempts to work through barriers to home practice, better documentation of such practice, and looking at other potential HPA and stress outcomes will be needed to clarify these issues.

We found no between group effects on adiposity or insulin sensitivity/resistance measures, although HOMA-IRI was reduced 15-21% in both groups (average within-group Δ = 0.3). This occurred despite a modest, non-significant increase in percent body fat in both groups, which we attribute to the short duration of the intervention and to its purposeful emphasis on a calorically non-restrictive philosophy. Since insulin resistance would be expected to worsen with an increase in adiposity, this finding suggests some portion of the intervention may benefit insulin sensitivity independent of adiposity. This could potentially be related to lowered cortisol exposure, since independent of intervention group, a decrease in serum cortisol predicted improvement in insulin sensitivity (Figure 4). Although imperfect as a measure of HPA activity, a single morning serum cortisol may still predict metabolic disease risk, as we have shown that higher morning serum cortisol is associated with the presence of metabolic syndrome cross-sectionally [28] and with deterioration of insulin sensitivity over time [29] in our study population. Larger scale, longer term studies will be needed to clarify the definitive effects of the IGI intervention on stress, stress biomarkers, and insulin sensitivity/resistance.

The major strength of this study is its strong randomized design to isolate the effects of IGI on stress reduction and health behavior change. The use of multidisciplinary outcome assessments – psychological, behavioral, biological – is another strength. Finally, applying this pilot intervention to a homogenous, high-risk adolescent population (obese, Latino teens in late puberty) helps fill a critical void in the adolescent obesity intervention literature. Limitations of the study primarily result from those inherent in a small pilot study of short duration that is designed primarily to determine intervention effect sizes on outcomes of interest, rather than to obtain definitive benefits. Though necessitated by the scope and primary aim of the study design, the lack of a true control group is an important limitation, and prevents full assessment of the intervention effects. While recognized as a strength, the homogenous study population is also a weakness in that findings cannot necessarily be generalized to all adolescent populations. Finally, though the reliance on self-report behavioral outcomes with their inherent risk of reporting biases is a limitation of our findings, we have used these dietary and activity measures in several prior obesity studies in youth, showing that they are valid and sensitive to change [52,73].

## Conclusions

The Imagine HEALTH intervention for obese Latino adolescents, both its didactic curriculum and its use of guided imagery, was acceptable to the study population. Improvements in physical activity following guided imagery support the role of non-cognitive, mind-body approaches to promoting health-behavior change in adolescent populations. Further investigations with a longer intervention and true, non-intervention control group will be needed to determine the full effects of the Imagine HEALTH intervention on stress, stress biomarkers, insulin resistance and adiposity outcomes.

## Competing interests

The co-author of “Intuitive Eating”, Elyse Resch, MS, RDN, is the spouse of the lead author and served as expert consultant on this project, designing the intuitive eating portion of the lifestyle intervention.

## Authors’ contributions

MJW conceived and designed the study, participated in data analysis and interpretation, and was primary author of original drafts and revisions of manuscript. CJL helped with study design, statistical consultation, generation of randomization schema, data analysis, and revisions of manuscript. QA performed enrollment and post-randomization group assignment of participants, data acquisition, drafting of methods section, and revisions of manuscript. KK performed data acquisition, drafting of dietary methods section, revisions of manuscript. EV performed data acquisition and analysis of dietary intake, drafting and revisions of manuscript. TA performed data analysis of intuitive eating data, drafting and revisions of manuscript. ZS was responsible for data base management, analysis of cortisol data, drafting and revisions of manuscript. MIG contributed to conception and study design, and revisions of manuscript. DSM participated in conception and study design, data analysis and interpretation, and drafting and revisions of manuscript. All authors read and approved the final manuscript.

## Pre-publication history

The pre-publication history for this paper can be accessed here:

http://www.biomedcentral.com/1472-6882/14/28/prepub
